# Coping during socio-political uncertainty

**DOI:** 10.3389/fpsyt.2023.1267603

**Published:** 2024-01-22

**Authors:** Myriam El Khoury-Malhame, Sandrella Bou Malhab, Roni Chaaya, Michel Sfeir, Samar El Khoury

**Affiliations:** ^1^Department of Social and Education Sciences, School of Arts and Sciences, Lebanese American University, Beirut, Lebanon; ^2^Department of Natural Sciences, School of Arts and Sciences, Lebanese American University, Beirut, Lebanon; ^3^Institut National de Sante Publique d’Epidemiologie Clinique et de Toxicologie, Liban (INSPECT-LB), Beirut, Lebanon; ^4^Department of Clinical Psychology, University of Mons, Mons, Belgium; ^5^Department of Sociology and Anthropology, Faculty of Letters and Human Sciences, Université Saint-Joseph, Beirut, Lebanon

**Keywords:** political unrest, coping, tolerance of uncertainty, well-being, anxiety, depression, COVID-19

## Abstract

**Introduction:**

Well-being of young adults is known to be compromised in times of significant changes, such as economic and political turmoil. This study focuses on university students in Lebanon during one of the most prominent social unrests of its modern history to determine potential understudied protective factors that would predict the youth capacity to strive.

**Methods:**

A sample of 489 university students were asked to fill an online survey including standardized questionnaires of wellbeing (WEMWBS), depression (PHQ-9), anxiety (HAM-A), intolerance of uncertainty (IUS-12), coping (Brief COPE) in addition to demographics and questions about their attitudes and future perspectives.

**Results:**

We found increased rates of mental distress, predominantly in women, with around 80% of the sample being highly intolerant to the uncertainty climate. Results unsurprisingly show that well-being negatively correlated with anxiety, depression and intolerance of uncertainty. Overall, mental distress was found to mediate the relation between uncertainty and wellbeing, and the relation between maladaptive coping and wellbeing. Students who were intolerant of uncertainty and who used maladaptive coping strategies were more likely develop anxiety and depression and subsequently report poorer wellbeing. Conversely, having adaptive strategies was directly linked to higher well-being.

**Discussion:**

In spite of increased distress, some university students managed to preserve their well-being within a climate of severe socio-political uprise. These findings suggest that modifying subjective experience of events and using soft skillset could alleviate young adults’ emotional distress in unstable societies.

## Highlights

- Young college students share major mental health struggles across political devise (pro-revolution or not) in times of severe political crisis and violent social uprise.
- Well-being of young college students is predicted by better tolerance of uncertainty, using adequate coping strategies and social support.
- Depression and anxiety moderate the relation between uncertainty and wellbeing as well as between maladaptive coping and wellbeing.
- Modifying subjective experiences of turmoil and promoting coping skillsets may improve longitudinal course of emotional distress and overall wellbeing in the active workforce.

## 1 Introduction

Political instability refers to an unexpected turn of events related to the collapse of a government due to growing struggles between various political parties either legally or by force (1). This concept comes in contradiction with political stability which is documented as a requirement for public and personal development attained by the management and balance of society’s stakeholders which aims at achieving common ideologies and goals (2). Political instability is known to influence individuals’ mental health as stress related to social and civil unrest is associated with elevated levels of both anxiety and depressive symptoms (3, 4). A recent systematic review aiming to update the World’s Health Organization estimates of the prevalence of mental health disorders in conflict settings found that one in five people in post-conflict situations struggled with mental health disorders such as: depression, anxiety, Post-traumatic Stress Disorder (PTSD), bipolar disorder and schizophrenia (5). Studies in Hong Kong for instance have found similar incidences during the country’s unrest period for depression, anxiety, and PTSD, in addition to reported self-perceived deterioration in emotional state (6, 7). Moreover, the prevalence of PTSD and depression after the Madrid train bombings in 2004 was around 2.3 and 8%, respectively. Worsened symptomology was associated with directly witnessing the event and predicted elevated levels of PTSD and depression (8). In a Lebanese study, stress and uncertainty of events were found to be negatively associated with well-being among Lebanese students (9). Young adults seem particularly vulnerable to such climate of tension, facing a sharp decline in the standard of living, security issues, interrupted schooling, and disruptions in essential services like water, electricity, waste removal, health care and social security (10).

The COVID-19 pandemic has further inflated the sense of instability and uncertainty with fear of the virus and of unexpected outcomes associated with increased levels of depression and anxiety as well as a plummeting of general well-being (11, 12). A systematic review documented extremely elevated rates of depression, anxiety, PTSD, and psychological distress globally, in countries like Spain, Turkey, Iran and the US (13). Case studies of this unprecedented viral infliction and its toll on human sanity report instances whereby individuals in Bangladesh or India committed suicide after they thought they were infected with the virus (14, 15). The pervasive fear related to COVID-19 uncertainty may be inflated by the mixed need to track rapidly evolving information about the deadly pandemic while attempting to avoiding watching the news and its evolution as these would increase one’s anxiety (16, 17). This echoes what Gigerenzer & Garcia-Retamero define as the regret of knowing, whereby individuals choose deliberate ignorance regarding a specific negative situation that may affect them ultimately leading them to lose control of their current situation (18).

Framed differently this fear of knowing/not knowing would qualify as intolerance of uncertainty which is a dispositional fear and a distress of the unknown or of future events and is known to interplay with emotional disturbances (19, 20). This intolerance of uncertainty initially studied with generalized anxiety disorder, is now associated with a wider range of disorders such as anxiety disorders, depression and PTSD (20–23). Intolerance of uncertainty was found to be negatively associated with well-being, with the fear of COVID-19 and rumination mediating the relationship (24). Furthermore, being uncertain about the risk of COVID-19 predicted higher levels of worry which in turn, predicted a low general well-being (25). These findings are in line with previous occurrences of earlier pandemics, where individuals’ intolerance of uncertainty and anxiety and depression levels were positively correlated, and inversely related to wellbeing (24, 26, 27). During the viral pandemic, individuals who could not tolerate the uncertainty related to the pandemic were more likely to experience negative emotions, manifested pathologically in the form of symptoms of anxiety, depression and fear and they were more likely to feel threatened by the uncertainty of the events (28). Seeking more information about the topic to address the intolerance of uncertainty in turn increases the negative emotions and worries generated by the unstable situation (28, 29). The association between intolerance of uncertainty and worry has long been examined (30, 31), as worry was shown to mediate the relationship between intolerance of uncertainty and depression (32).

Although instability, whether political or medical, is known to increase mental distress in general and in young adults in particular (33, 34), some individuals seem less affected by its devastating effects. Evidence suggests that individual using adaptive coping strategies have higher levels of overall well-being. For instance, a systematic review has shown that nurses who used adaptive coping strategies had lower levels of burnout and exhaustion (35). Using an approach coping strategy was previously found to be positively associated with well-being as rationally decreasing stress levels may warrant individuals to feel more in control of a catastrophic collective trauma (36–38). Intolerance of uncertainty has been associated with less positive coping style (39).

### 1.1 Context and current study

Lebanon is a small middle eastern country plowing under accumulating adversities including the devastating COVID-19 pandemic, the unprecedented socio-political unrest with a million people marching the street, the dire economic situation with the worst global financial crisis of the past 150 years (40) with a massive devaluation of the local currency and the highest influx of Syrian refugees (12). In fact, just prior to the pandemic, the October 17 uprise was marked by violent polarized political devise, demanding the demise of the ruling class and escalating a climate of tension and severe verbal aggression and social media trolling between those in support of attacking all political figures in place vs. those favoring classical political parties and demanding a smoother transition. With decades of civil war and political assassinations, and lack of governmental and institutionalized improvements, the Lebanese residents have been experiencing many direct threats to their basic needs and livelihoods in addition to major uncertainties related to their future, pushing many young active educated citizens toward immigration (41–43). With the ongoing political problems punctuating the Lebanese history, uncertainty and instability have become the only constant in Lebanon (9). Lebanese adults had reported drastic drop in their overall well-being (37) and associated mental health struggles, with the financial deterioration and the pandemic in that order (44, 45).

**Figure 1:**
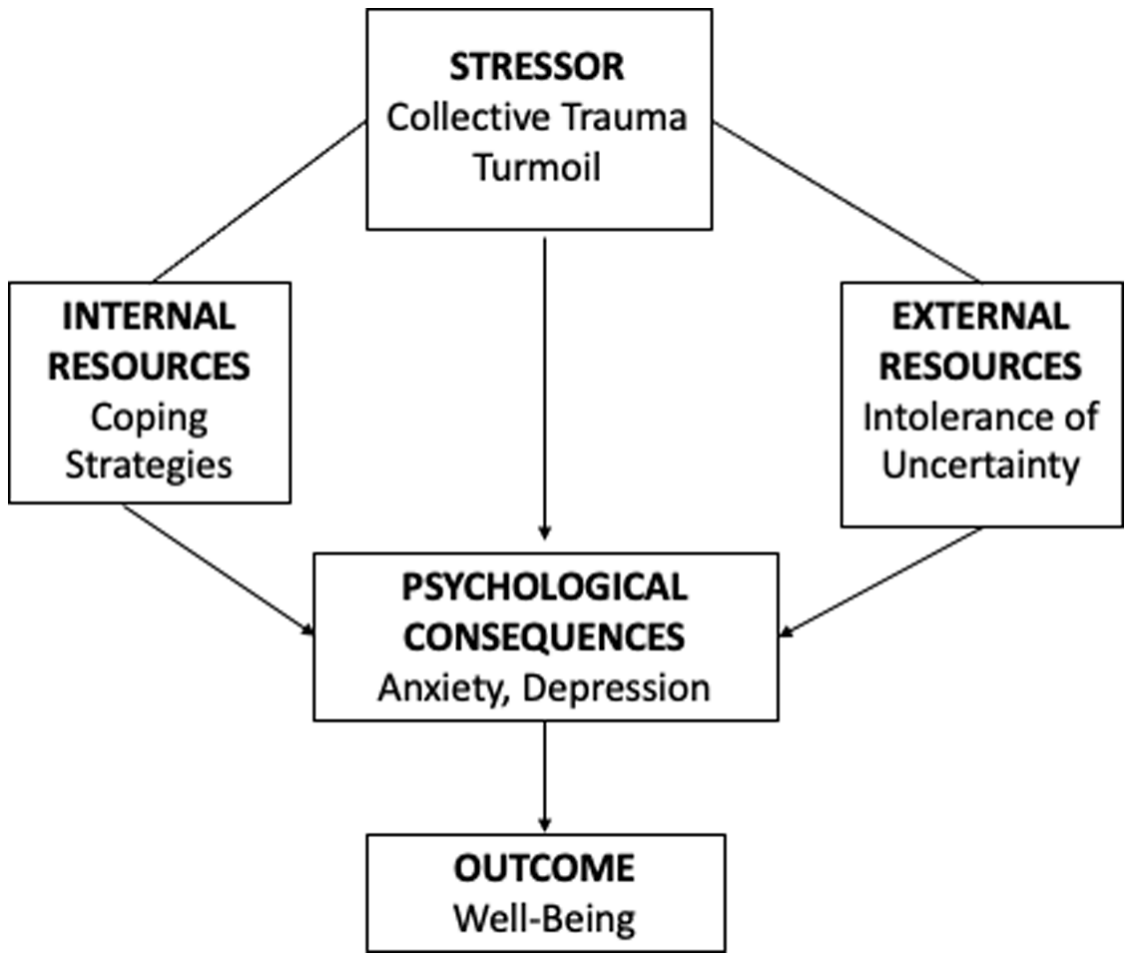
Study model.

The conceptual model used is the Seiffe-Krenke Developmental model for young adults represented below (Figure 1), used as a guiding framework for studies on youth and previously validated in the Lebanese context (9). The model presents a theoretical conceptualization based on empirical research analyzing normative young adults’ developmental goals and differential adjustment to change to build a healthy overall transition to adulthood.

The goal of the current paper was to assess the potential mediating effect of anxiety and depression on wellbeing of university students considering their tolerance of uncertainty and coping mechanisms, during the accumulating challenges causing an unstable socio-political situation in Lebanon. We hypothesized that under those critical conditions, students with better coping strategies and a higher sense of tolerance of the unknown would display improved well-being compared to others. This is particularly relevant in this age group as youth are altogether a vulnerable group to distress but also the country’s hope post-collective trauma to actively contribute to the revival process.

## 2 Materials and methods

### 2.1 Study design and participants

In this cross-sectional study, young Lebanese university students were asked to fill an online questionnaire aimed at assessing wellbeing in times of major political turmoil. Demographic data included gender, education level (academic year input), and financial status (bracket segments) in addition to questions about supporting the uprise or not (yes/no), planning to stay in Lebanon after graduation or not (yes/no), believing the university provided skills to navigate the future or not (on a Likert scale from 1 to 4). After providing informed consent, individuals filled out the questionnaire online and submitted it once completed. The final sample included 489 individuals older than 18 years and currently enrolled in a university in Lebanon (bachelor and masters students) with 224 males (45.8%).

### 2.2 Procedure

The study was conducted according to the Declaration of Helsinki. After obtaining the ethical approval from the IRB board at the Lebanese American University. An online link was sent to university students by convenience sampling and sharing the link on social media accounts (WhatsApp, Facebook, LinkedIn and Instagram). Participants approved the informed consent before filling the survey.

### 2.3 Instruments

#### 2.3.1 The Warwick-Edinburgh mental well-being scale

It is a scale of 14 positively worded items for assessing a population’s mental well-being (46). The WEMWBS is suitable for adults aged 16 and above.

#### 2.3.2 Patient health questionnaire-9

It was used to measure depression. It is a self-rated 9 items measure that assesses the severity of depressive symptoms in individuals aged 18 and older (47, 48). Each item is rated on a 4-point scale (0 = Not at all; 1 = Several days; 2 = More than half the days; and 3 = Nearly every day). The total score can range from 0 to 27, with higher scores indicating greater severity of depression (49); such as 5–9 are indicative of mild depressive symptoms, 10–14, moderate, 15–19 moderately severe and above 20 severe symptoms.

#### 2.3.3 Hamilton anxiety rating scale

It is one of the first rating scales developed to measure the severity of anxiety symptoms (50). It consists of 14 items, each probing a given symptom. It measures both mental and physical distress. Each item is scored on a scale from 0 (Not present) to 4 (Severe), with a total score range of 0–56, where <17 indicates mild severity, 18–24 mild to moderate severity and 25–30 moderate to severe symptomatology.

#### 2.3.4 Intolerance of uncertainty

It includes 12 items relating to the idea that uncertainty is unacceptable, reflects badly on a person, and leads to frustration, stress, and the inability to take action (51). Participants rate items on a 5-point Likert scale ranging from 1 = “not at all characteristic of me” to 5 = “entirely characteristic of me.” Higher scores represent higher intolerance of uncertainty.

#### 2.3.5 The brief COPE

The Brief COPE is a brief version of the COPE (Coping Orientation to Problems Experienced) (52). It is a self-report questionnaire developed to assess a broad range of coping responses. The instrument typically consists of 28 items on a Likert scale ranging from 0 = I have not been doing this at all to 3 = I have been doing this a lot. For the purpose of this study, the scale was divided into 2 main factors based on psychometric properties analyses included as Supplementary Tables as well as in parallel with an advanced recent finding of a validation study in 30 countries including Lebanon suggesting improved predictability of this factor analysis (Sanchez et al., submitted). The only subscales loading and retained include:

F1: Adaptive Coping: Active Coping, Positive Reframing, Planning, Acceptance, Emotional Support, Instrumental Support.

F2: Maladaptive Coping: Negative Attitudes such as Denial, Behavioral Disengagement, Self-Blame and Substance Use.

### 2.4 Statistical analysis

Data were analyzed on SPSS software version 25. A descriptive analysis was performed using absolute frequencies and percentages for categorical variables and means and standard deviations (SD) for quantitative measures.

Construct validity was performed using the rotated component matrix technique. The Kaiser-Meyer-Olkin measure of sampling adequacy and Bartlett’s test of sphericity were calculated to ensure the model’s adequacy. Factors with eigenvalues >1 were retained, and the scree plot method was used to determine the number of components to extract (53). Only items with factor loading >0.4 were considered. Moreover, the internal consistency of the scales was assessed using Cronbach’s alpha. Data is tabulated in Supplementary Tables S1–S6.

For bivariate analysis, Pearson correlation was calculated for continuous variables.

A mediation analysis using PROCESS version 4.2 was run to measure the mediating effect (M) of anxiety and depression on wellbeing (Y) as the dependent variable and intolerance of uncertainty, maladaptive coping and adaptive coping as separate independent variables (X). Pathway A determined the regression coefficient for the effect of X on M. Pathway B examined the association between M with Y, independent of X, and Pathway C estimated the total and direct effect of X on Y. Pathway AB calculated the indirect intervention effects. The estimated 95%CI by bootstrapping of the indirect effect of X on Y was performed to calculate the significance of the mediation effect. The covariates that were included in the mediation model were those that showed significant associations in the bivariate analysis. A *p*-value less than 0.05 was considered significant. Data is tabulated in Supplementary Tables S7–S9.

### 2.5 Factor analysis

A factor analysis using the rotated component matrix technique was used to test the construct validity of the used scales and ensure the model’s adequacy. All items of the scales could be extracted from the list, and none of them was removed because of over-correlation (*r* > 0.9), had a low loading on factors (<0.4), or a low communality (<0.4), except for the coping scale factors 20, 21, 24. The details of the Kaiser-Meyer-Olkin measure of sampling adequacy, Bartlett’s test of sphericity and the total Cronbach alpha are found in Supplementary Tables. Increased score indicates higher levels of anxiety, depression, wellbeing, intolerance of uncertainty and coping.

## 3 Results

### 3.1 Socio-demographics and sample characteristics

The demographic data for the 489 participants who were included in this study is illustrated in Table 1. Descriptive data shows that more than 75% of the young students support the massive uprise, and 50–60% have plans to study or work abroad.

**Table 1:** Sociodemographic characteristics of the participants.

*N* = 489	
Variable	*N* (%)
**Gender**	
Male	224 (45.8%)
Female	265 (54.2%)
**Academic year**	
Freshman, Sophomore or Junior	223 (45.8%)
Senior	264 (54.2%)
**Do you support the Oct. 17 uprise?**	
Yes	372 (76.1%)
No	117 (23.9%)
**If you have plans for continuing your studies, where would you choose?**	
Private university, Lebanon	163 (36.1%)
Public university, Lebanon	44 (9.8%)
Abroad	213 (47.2%)
Other	13 (2.9%)
**If you have plans to start working, where would you choose?**	
Lebanon	181 (40.1%)
Abroad	263 (58.3%)

PS: Some values do not add up to the total N because of missing values.

An additional chi-square analysis showed that those who supported the socio-political uprise were more likely than others to seek opportunities abroad than to stay in Lebanon, whether for education (*X*^2^ = 16.781, *p* < 0.001) or work (*X*^2^ = 16.781, *p* < 0.001).

### 3.2 Means and percentages of continuous variables

The mean values of the continuous psychological variables are highlighted in Table 2.

**Table 2:** Values of different continuous psychological variables.

Scale	Mean ± SD	Cronbach alpha	%Low levels	%Severe/High levels
WEMWBS	45.38 ± 10.76	0.902	35.5%	9%
IUS-12	35.42 ± 10.69	0.874	18%	82%
HAM-A	18.45 ± 10.31	0.876	39.5%	27.5%
PHQ-9	11.73 ± 10.69	0.844	12.9%	10.4%
Coping	53.35 ± 13.18	0.844		

The table represents the mean and Standard deviation (SD) as well as Cronbach alpha and % of participants scoring low and high on the following scales: well-being (WEMWBS), intolerance of uncertainty (IUS-12), anxiety (HAM-A), depression (PHQ9) and Coping factors.

Among the participants, only around 9% scored high on wellbeing, whereas 82% of participants had high intolerance of uncertainty. Additionally, 27.5% of participants scored above cut-off for severe anxiety and 10.4% for severe depressive symptoms.

### 3.3 Bivariate correlations

Results show an unsurprising negative correlation between well-being on one hand and mental distress including anxiety and depression on the other. Additionally, well-being positively correlated with and adaptive coping factors of the Brief Cope scale but negatively correlated with maladaptive coping (Table 3).

**Table 3:** Bivariate analysis of continuous variables associated with the total well-being score.

Variable	Correlation coefficient	*p*
IUS-12	−0.041	0.386
HAM-A	−0.296	**<0.001**
PHQ-9	−0.456	**<0.001**
Adaptive coping	0.430	**<0.001**
Maladaptive coping	−0.116	**0.011**

The table represents the Pearson correlation scores and *p*-values for variables correlating well-being (WEMWBS): intolerance of uncertainty (IUS-12), anxiety (HAM-A), depression (PHQ9) as well as adaptive and maladaptive coping factors subsets. Significant *p*-values <0.05 are represented in bold.

We also found a positive association between well-being and confidence in one’s skills upon graduation (*r* = 0.38; *p* < 0.001), as well as a strong positive association between depression and anxiety (*r* = 0.708; *p* < 0.001). In turn hose aforementioned variables positively correlated with intolerance of uncertainty (*r* = 0.280 and *r* = 0.365, p < 0.001 respectively) and with maladaptive coping (*r* = 0.455 and *r* = 0435; p < 0.001 respectively).

Males had higher levels of well-being compared to females (*p* < 0.05). Moreover, when compared to males, females had higher scores of anxiety and depression symptoms (Table 4).

**Table 4:** Bivariate analysis of categorical variables associated with the scores of the psychological variables.

Dependent variable	Independent variable	Mean ± SD	*p*	Effect size
Well-being	Gender		**0.024**	0.017
	Male	46.77 ± 11.62		
	Female	43.91 ± 9.69		
Anxiety	Gender		**0.004**	0.026
	Male	16.82 ± 10.19		
	Female	20.19 ± 10.16		
Depression	Gender		**0.003**	0.027
	Male	10.73 ± 5.95		
	Female	12.74 ± 6.04		

The table represents the bivariate analysis of wellbeing, anxiety and depression across gender. Significant p-values <0.05 are represented in bold.

No such differences were found for those in support or not of the uprise.

### 3.4 Mediation models

The below diagrams illustrate the mediation analysis conducted on the relation between intolerance of uncertainty scores, coping style and wellbeing.

**Figure 2:**
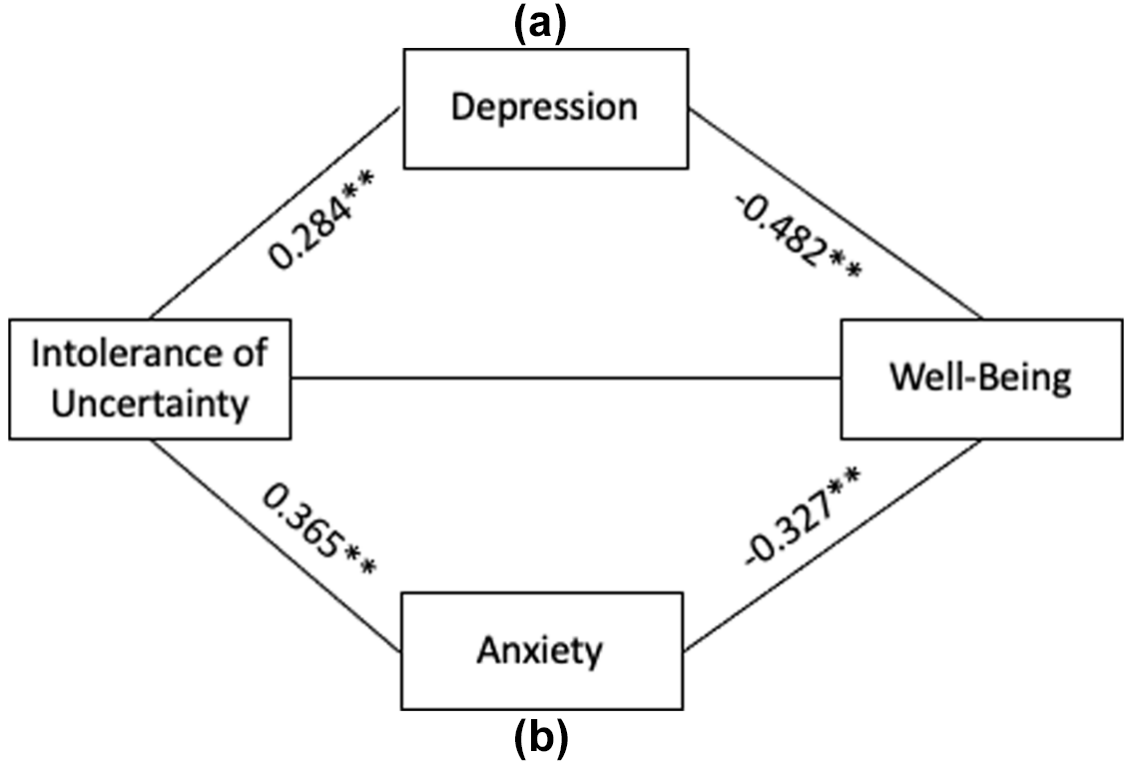
Mediation models of the impact of intolerance of uncertainty on well-being (a) Depression (b) Anxiety. Both models are significant with confidence intervals not containing 0. All values represent significant Beta values for the causal interaction with *p < 0.05 and **p < 0.001. Pathways (a) and (b) are independent and separate but represented in the same diagram for ease of reading.

We found that anxiety and depression significantly mediated the impact of intolerance of uncertainty on well-being. There was no significant direct effect of intolerance of uncertainty on wellbeing (Figure 2). This means that symptoms of psychopathology are elevated by intolerance of the climate of overall instability and in turn cause the drop in levels of well-being in university students. Relevant Tables can be found in Supplement material.

**Figure 3:**
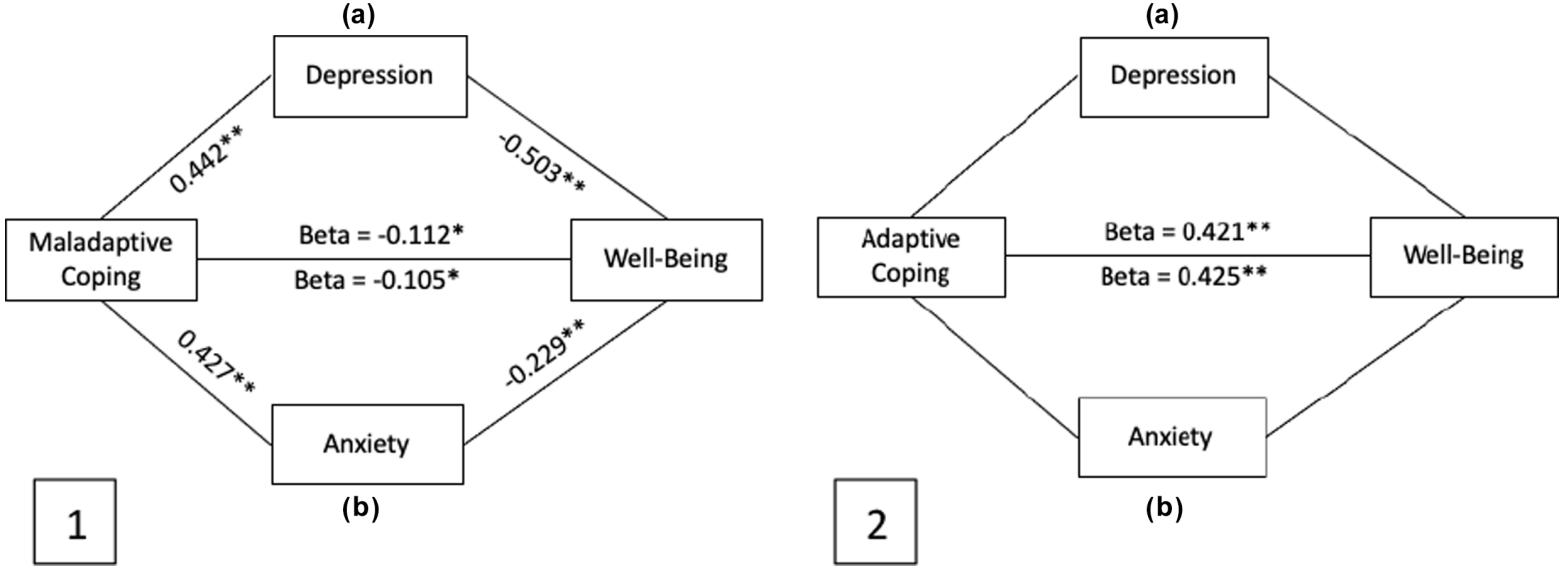
2.1. Mediation models of the impact of maladaptive coping on well-being by (a) Depression (b) Anxiety. Both models are significant with confidence intervals not containing 0. All values represent significant Beta values for the causal interactions with *p < 0.05 and **p < 0.001. 2.2. Mediation models of the impact of adaptive coping on well-being by (a) Depression (b) Anxiety. Neither model is significant. Only adaptive coping directly impacts well-being with significant Beta values for the causal interactions with *p < 0.05 and **p < 0.001. In each model pathways (a) and (b) are independent and separate but represented in the same diagram for ease of reading.

Figure 3 also represents the mediation analysis conducted on the impact of maladaptive and adaptive coping scores on well-being. Both anxiety and depression significantly mediated the association between the maladaptive coping and wellbeing. Maladaptive coping had a partial effect on wellbeing. However, adaptive coping had a positive effect on wellbeing, but anxiety and depression had no mediating effect. Relevant Tables can be found in Supplement material.

## 4 Discussion

This study investigated the mediating effect of anxiety and depression on the well-being of young university students in relation to intolerance of uncertainty and coping resources in times of severe socio-political unrest in Lebanon. We show that, amidst the massive ongoing unprecedented turmoil, those who better tolerate the climate of uncertainty, have lower levels of psychopathological symptoms and subsequently report higher levels well-being. Also, regardless of mental health status, resorting to adaptive coping strategies not maladaptive ones seems to improve overall well-being.

First and foremost, the findings illustrate high levels of anxiety and depression alongside impoverished well-being of young students faced with the accumulating challenges. The massive political instability and violent protests indeed contribute to a dramatic increase in uncertainty about the future among young individuals with prospects of graduating and joining the workforce. This translates in severe symptoms of anxiety and worry for 1/3 of the participants. The wellbeing of this age group, known to be vulnerable to psychopathology, also decreases (54). Recent findings had indeed documented that successive Lebanese crises have led to an incremental rise of citizens’ psychological distress, especially among young adults, worsening their overall well-being (55). The weighing climate of uncertainty spared no political party and the mental health bill for socio-political and financial changes was pricey across the acute political devise, especially with the underlying COVID-19 viral threat. We indeed found no differences in occurrences of mental distress or well-being among those who were in favor of the revolutionary demands v/s those who were not. Surprisingly however, those demanding the high-priced change in structural foundations of the country were more likely than others to plan their departure from the country they were protesting to see improve. It could be that those young people have particular mistrust of the failed state and economy (56, 57), further aggravating their intolerance of uncertainty about their futures. They might also be predominantly disillusioned with their capacities to ignite sustainable change in a post-war country with the same war lords turned political elites since the early 1990s (58) and would thus prefer to flee the stressful uncontrollable environment.

Our results further show that mental distress was a significant mediator of the association between intolerance of uncertainty and deteriorating well-being in university students as those with lower tolerance to the unpredictable nature of the traumatic circumstances had higher levels of both depression and anxiety symptoms, consequently leading to poorer levels of overall well-being. This corroborates previous solid results showing an inverse relationship between well-being and psychopathology, namely depression and anxiety (59), with poor quality of life in turn exacerbating people’s mental distress. Previous studies had also shown that depression and anxiety mediate the impact of frontal brain white matter integrity, involved in emotional regulation capacity and quality of life in young adults (60). As such, since acting on environmental challenges causing uncertainty, whether viral or political, is beyond the realm of clinicians and public health providers, developing strategies to address psychopathology could improve well-being in unstable societies (61).

Among those strategies, adaptive coping factors were shown to play a direct protective role of overall well-being. Students with in adaptive coping skillsets like positive reframing, planning and acceptance, and who rely on soliciting emotional and instrumental support, tend to have higher levels of wellbeing. Those positive coping strategies, support-seeking behaviors, problem-focused positive reframing and emotional regulation, are all well documented ways to improve levels of psychological well-being and significantly lower distress (62). On the other hand, maladaptive coping is associated with higher levels of mental struggles (63) which in turn lower psychological wellbeing (62). The study by Meng and D'Arcy (62) in fact suggests that people with higher levels of distress tend to resort to maladaptive coping strategies such as denial, substance abuse and behavioral disengagement, which results in systematically worsening their mental states. Thus, in spite of massive political and economic instability, those who manage to resort to adaptive ways of dealing with their demanding environments have better outcomes in regard to their well-being (62).

Most importantly our results indicate that the adequate coping strategies taken together with tolerating sociopolitical and economic instability, as well as being a male and believing in one’s acquired skills via university education were found to be correlates of well-being in those dire times. These altogether suggest on one hand the presence of inter-individual variability facing adversity and one the other, that individual perception of adversity as well as personal skills, rather than the mere intensity of adversity itself, would predict the levels of adjustment and subsequent well-being of individuals, even in the context of major political uprise and global pandemic. Students who are able to tolerate the uncertainty triggered by unprecedented collective changes, and who perceive that their university years have equipped them with knowledge and skills to find suitable jobs post-graduation in spite on the crisis, report feeling better about themselves. Also, those who have developed personal resources such as adaptive, and support-seeking coping seem to better manage their well-being compared to their fellow students. These results resonate with very recent findings by Danese et al. (64) emphasizing that subjective assessments of major events, as opposed to the traditional sheer objective evaluation of those events, should be considered when investigating the determinants of emotional disorders in adults following adverse exposures in childhood. Within the same optic, Satici et al. (24) had illustrated a direct effect of intolerance of uncertainty on well-being mediated by rumination and fear (24). It would seem that the Lebanese socio-political turmoil is not a fatality on the mental health of young adults, and, endurance of uncertainty, in addition to resilience and social support are significant determinants of their well-being in such dire times (9). This could be explained by findings that positive self-perception is related to positive psychological wellbeing and that improving the confidence and self-esteem of individuals can be effective in improving their wellbeing, regardless of situational environmental factors alone (65).

It is noteworthy that in our model gender was a statistical predictor of wellbeing. Males seem to navigate those challenges more adaptively and with more comfort than females as per the self-determination theory due to fulfillment of needs of autonomy and competency, whereas female exhibit lower psychological wellbeing (66). According to the authors, a sense of proficiency does indeed equip young adults with an improved capacity to deal with uncertainty and do better under stress. In addition, there could to be a relevant cultural middle eastern aspect to this gendered discrepancy, that might be due to the nature of the patriarchal society in Lebanon where males may receive more opportunities and privileges than females and are thus made to feel empowered to act upon their environment (67).

### 4.1 Limitations

This study investigated the wellbeing of 489 Lebanese undergraduate students and findings should be very cautiously generalized to the Lebanese population at large. The snowball sampling technique used to recruit the sample has limitations in and of itself which might additionally bias the representativeness of the results. On the other hand, some confounding variables affecting well-being may have been left out in our analyses. These include for instance the effects of lockdown and social distancing on the one’s well-being and the subsequent assessment of his/her future. They also include potential contamination, or exposure due to being in healthcare professions. Other confounds may be participants’ religious beliefs linked to life satisfaction and well-being (37). Future research should focus on increasing sample size and diversifying the sample population to better represent national standards and potential cross-cultural ones. Longitudinal studies may be needed to better study the long-term effect of traversing socio-political unrest on the well-being and coping capacities of young students and tentatively better understand the causality between the independent variables and well-being.

### 4.2 Clinical implications

Our findings suggest gendered vulnerability of university students during political instability as females score higher than males on depression, anxiety and lower on wellbeing. It further underlies the importance of focusing on individuals’ subjective perception of the events as well as using different adaptive coping strategies to bolster the deleterious effects of uncertainty and aid in enhancing well-being of undergraduates. It could be that in uncertain times, having an authority attempting to relativize the situation and promote coping strategies such as support-seeking mechanisms would aid students to preserve their well-being in general and more specifically in the Lebanese context. Additionally, since public health providers and educators have no or limited impact on the accumulating socio-political, economic and viral threats and their subsequently induced uncertainty, we suggest that they could alternatively promote targeted programs to improve youth wellbeing by addressing psychopathology symptoms of depression and anxiety; as these symptoms were shown to moderate the impact of subjective experience of uncertainty on overall wellbeing. This could be done by helping at-risk young adults manage their distress and regulate their emotional intelligence (37). Those programs would also contribute to increase their documented ability to further grow and become more resilient post-trauma (44).

## 5 Conclusion

This study investigated the mediating effect of depression and anxiety on the impact of intolerance of uncertainty, use of adaptive or maladaptive coping strategies on well-being in times of political unrest. It highlights subjective individual differences and personal competences and therefore adds to the accumulating literature on the determinants of well-being across Lebanese undergraduate students faced with collective traumas and social as well as medical threats and instabilities. Further exploring personal subjective use of resources, cognitive, emotional and behavioral skills could help increase tolerance of uncertainty and safeguard well-being. In a society very much attuned to spiritual and religious practices, exploring the relationship between spirituality, well-being and levels of intolerance of uncertainty could also offer scalable interventions.

## Data availability statement

The raw data supporting the conclusions of this article will be made available by the authors, without undue reservation.

## Ethics statement

The studies involving humans were approved by Institutional Review Board at the Lebanese American University. The studies were conducted in accordance with the local legislation and institutional requirements. The participants provided their written informed consent to participate in this study.

## Author contributions

ME: Conceptualization, Methodology. Investigation, Writing – original draft. SB: Formal analysis, Resources, Software, Visualization, Writing – review & editing. RC: Investigation, Resources, Writing – original draft. MS: Investigation, Writing – original draft. SE: Methodology, Project administration, Supervision, Writing – review & editing.
