# Supplementary material for: Coping during socio-political uncertainty
Source: Front Psychiatry. 2024 Jan 22;14:1267603. doi: 10.3389/fpsyt.2023.1267603 (PMC10839968; doi:10.3389/fpsyt.2023.1267603)
Supplement: Supplementary file 1 [file Table_1.DOCX]

Supplement Tables 1, 2, 3, 4 and 5 detail the psychometric properties of the used scales

| **Supplement Table 1. Rotated component matrix of the modified Hamilton scale for anxiety (HAM-A)** | | | | | |
| --- | --- | --- | --- | --- | --- |
| Question to what extent do you fell the following | **Item** | **Factor 1 Physiological Symptoms** | **Factor 2 Cognitive Emotional Symptoms** | **Factor 3 Fear Expression** | **H2 communalities** |
| **Cardiovascular**. Tachycardia, palpitations, pain in chest, throbbing of vessels, fainting feelings, missing beat | 9 | 0.827 |  |  | 0.674 |
| **Gastrointestinal Symptoms.** Difficulty in swallowing, wind abdominal pain, burning sensations, abdominal fullness, nausea, vomiting, borborygmi, looseness of bowels, loss of weight, constipation | 11 | 0.790 |  |  | 0.654 |
| **Respiratory**. Pressure or constriction in chest, choking feelings, sighing, dyspnea. | 10 | 0.783 |  |  | 0.671 |
| **Somatic** (muscular). Pains and aches, twitching, stiffness, myoclonic jerks, grinding of teeth, unsteady voice, increased muscular tone | 7 | 0.645 |  |  | 0.530 |
| **Genitourinary Symptoms**. Frequency of micturition, urgency of micturition, amenorrhea, menorrhagia, development of frigidity, premature ejaculation, loss of libido, impotence. | 12 | 0.626 |  |  | 0.715 |
| **Somatic** (sensory). Tinnitus, blurring of vision, hot and cold flushes, feelings of weakness, pricking sensation. | 8 | 0.533 |  |  | 0.559 |
| **Anxious mood.** Worries, anticipation of the worst, fearful anticipation, irritability. | 1 |  | 0.818 |  | 0.657 |
| **Tension**. Feelings of tension, fatigability, startle response, moved to tears  easily, trembling, feelings of restlessness, inability to relax. | 2 |  | 0.791 |  | 0.637 |
| **Intellectual**. Difficulty in concentration, poor memory. | 5 |  | 0.508 |  | 0.505 |
| **Insomnia**. Difficulty in falling asleep, broken sleep, unsatisfying sleep and fatigue on waking, dreams, nightmares, night terrors. | 4 |  | 0.502 |  | 0.425 |
| **Depressed Mood.** Loss of interest, lack of pleasure in hobbies, depression, early waking, diurnal swing. | 6 |  | 0.433 |  | 0.403 |
| **Behavior**. Fidgeting, restlessness or pacing, tremor of hands, furrowed brow,  strained face, sighing or rapid respiration, facial pallor, swallowing,  etc | 14 |  |  | 0.718 | 0.554 |
| **Autonomic symptoms**. Dry mouth, flushing, pallor, tendency to sweat, giddiness, tension headache, raising of hair. | 13 |  |  | 0.612 | 0.725 |
| **Fears.** Of dark, of strangers, of being left alone, of animals, of traffic, of  crowds. | 3 |  |  | 0.579 | 0.505 |
| **Total variance explained** |  | 39.68% | 11.57% | 7.42% | 58.66% |
| **Cronbach alpha = 0.876** | | | | | |
| **Kaiser-Meyer-Olkin (KMO) = 0.897** | | | | | |
| **Bartlett’s test of sphericity p < 0.001** | | | | | |

| **Supplement Table2. Rotated component matrix of the modified scale for depression (PHQ-9)** | | | | |
| --- | --- | --- | --- | --- |
| Question: how often you feel the following | **Item** | **Factor 1 Core Symptoms** | **Factor 2 Cognitive and Psychomotor Symptoms** | **H2 communalities** |
| Feeling tired or having little energy? | 4 | 0.800 |  | 0.642 |
| Trouble falling or staying asleep, or sleeping too much? | 3 | 0.792 |  | 0.570 |
| Feeling down, depressed, or hopeless? | 2 | 0.756 |  | 0.628 |
| Feeling bad about yourself — or that you are a failure or have let yourself or your family down? | 6 | 0.723 |  | 0.606 |
| Poor appetite or overeating? | 5 | 0.714 |  | 0.549 |
| Little interest or pleasure in doing things? | 1 | 0.497 |  | 0.191 |
| Thoughts that you would be better off dead, or thoughts of hurting yourself in some way? | 9 |  | 0.967 | 0.742 |
| Moving or speaking so slowly that other people could have noticed? Or so fidgety or restless that you have been moving a lot more than usual? | 8 |  | 0.758 | 0.651 |
| Trouble concentrating on things, such as reading the newspaper or watching television? | 7 |  | 0.587 | 0.568 |
| **Total variance explained** |  | 45.34% | 11.84% | 57.18% |
| **Cronbach alpha = 0.844** | | | | |
| **Kaiser-Meyer-Olkin (KMO) = 0.872** | | | | |
| **Bartlett’s test of sphericity p < 0.001** | | | | |

| **Supplement Table 3. 4 Rotated component matrix of the modified scale for wellbeing (WEMWBS)** | | | | |
| --- | --- | --- | --- | --- |
| Question: how you have been feeling for the past 2 weeks | **Item** | **Factor 1 Psychological Wellbeing** | **Factor 2 Positive Mental Wellbeing** | **H2 communalities** |
| I’ve been feeling confident | 10 | 0.842 |  | 0.638 |
| I’ve been able to make up my own mind about things | 11 | 0.841 |  | 0.665 |
| I’ve been feeling good about myself | 8 | 0.803 |  | 0.672 |
| I’ve been feeling loved | 12 | 0.778 |  | 0.482 |
| I’ve been thinking clearly | 7 | 0.690 |  | 0.531 |
| I’ve been interested in new things | 13 | 0.673 |  | 0.456 |
| I’ve been dealing with problems well | 6 | 0.664 |  | 0.442 |
| I’ve been feeling cheerful | 14 | 0.563 |  | 0.660 |
| I’ve been feeling close to other people | 9 | 0.467 |  | 0.419 |
| I’ve had energy to spare | 5 |  | 0.845 | 0.595 |
| I’ve been interested in other people | 4 |  | 0.809 | 0.552 |
| I’ve been feeling useful | 2 |  | 0.671 | 0.496 |
| I’ve been feeling optimistic about the future | 1 |  | 0.638 | 0.422 |
| I’ve been feeling relaxed | 3 |  | 0.615 | 0.568 |
| **Total variance explained** |  | 44.89% | 9.38% | 54.28% |
| **Cronbach alpha = 0.902** | | | | |
| **Kaiser-Meyer-Olkin (KMO) = 0.916** | | | | |
| **Bartlett’s test of sphericity p < 0.001** | | | | |

| **Supplement Table 4. Rotated component matrix for the modified coping scale (Brief Cope)** | | | | | | | | | |
| --- | --- | --- | --- | --- | --- | --- | --- | --- | --- |
| Question | **Item** | **Factor 1 negative attitude** | **Factor 2 active coping** | **Factor 3 positive attitude** | **Factor 4 spirituality** | **Factor 5 support seeking** | **Factor 6 substance abuse** | **Factor 7 getting busy** | **H2 communalities** |
| I have been refusing to believe that it has happened | 8 | 0.844 |  |  |  |  |  |  | 0.724 |
| I have been saying to myself that this is not real | 3 | 0.723 |  |  |  |  |  |  | 0.574 |
| I have been giving up trying to deal with it | 6 | 0.665 |  |  |  |  |  |  | 0.541 |
| I have been saying things to let my unpleasant feelings escape | 9 | 0.594 |  |  |  |  |  |  | 0.472 |
| I have been blaming myself for things that happened | 26 | 0.560 |  |  |  |  |  |  | 0.504 |
| I gave up the attempt to cope | 17 | 0.540 |  |  |  |  |  |  | 0.517 |
| I have been criticizing myself | 16 | 0.480 |  |  |  |  |  |  | 0.529 |
| I have been taking action to make the situation better | 7 |  | 0.844 |  |  |  |  |  | 0.623 |
| I have been concentrating my efforts on doing something about the situation I am in | 2 |  | 0.810 |  |  |  |  |  | 0.713 |
| I have been trying to come up with a strategy about what to do | 14 |  | 0.752 |  |  |  |  |  | 0.609 |
| I have been thinking hard about what steps to take | 25 |  | 0.542 |  |  |  |  |  | 0.562 |
| I have been trying to see it in a different light, to make it seem more positive | 12 |  | 0.403 |  |  |  |  |  | 0.539 |
| I have been making jokes about it | 19 |  |  | 0.910 |  |  |  |  | 0.713 |
| I have been making fun of the situation | 28 |  |  | 0.847 |  |  |  |  | 0.636 |
| I have been looking for something good in what is happening | 18 |  |  | 0.451 |  |  |  |  | 0.581 |
| I have been trying to find comfort in my religion or spiritual beliefs | 22 |  |  |  | 0.974 |  |  |  | 0.780 |
| I have been praying or meditation | 27 |  |  |  | 0.917 |  |  |  | 0.730 |
| I have been getting emotional support from others | 5 |  |  |  |  | 0.873 |  |  | 0.739 |
| I have been getting comfort and understanding from someone | 15 |  |  |  |  | 0.661 |  |  | 0.630 |
| I have been trying to get advice or help from other people about what to do | 23 |  |  |  |  | 0.571 |  |  | 0.645 |
| I have been using alcohol to make me feel better | 4 |  |  |  |  |  | 0.843 |  | 0.807 |
| I have been using alcohol or drugs to get me through it | 11 |  |  |  |  |  | 0.822 |  | 0.773 |
| I have been turning to work or other activities to take my mind off things | 1 |  |  |  |  |  |  | 0.740 | 0.566 |
| **Total variance explained** |  | 22.59% | 13.86% | 6.47% | 5.83% | 4.32% | 4.08% | 3.93% | 61.08% |
| **Cronbach alpha = 0.844** | | | | | | | | | |
| **Kaiser-Meyer-Olkin (KMO) = 0.829** | | | | | | | | | |
| **Bartlett’s test of sphericity p < 0.001** | | | | | | | | | |
| **The coping scale covered 2 facets of coping:**  **Maladaptive coping includes factors 1 and 6. Cronbach alpha = 0.819**  **Adaptive coping includes factors 2; 3; 4; 5 and 7. Cronbach alpha = 0.837** | | | | | | | | | |

| **Supplement Table 5. Rotated component matrix of the modified scale for intolerance of uncertainty (IUS-12)** | | | | |
| --- | --- | --- | --- | --- |
| Question: how much you agree with each item | **Item** | **Factor 1 Prospective Anxiety** | **Factor 2 Inhibitory Anxiety** | **H2 communalities** |
| One should always look ahead so as to avoid surprises. | 4 | 0.927 |  | 0.617 |
| I should be able to organize everything in advance. | 11 | 0.770 |  | 0.510 |
| It frustrates me not having all the information I need. | 2 | 0.603 |  | 0.456 |
| I always want to know what the future has in store for me. | 8 | 0.572 |  | 0.358 |
| Unforeseen events upset me greatly. | 1 | 0.497 |  | 0.393 |
| A small unforeseen event can spoil everything, even with the best of planning. | 5 | 0.482 |  | 0.531 |
| I must get away from all uncertain situations. | 12 | 0.446 |  | 0.472 |
| When it’s time to act, uncertainty paralyses me. | 6 |  | 0.898 | 0.701 |
| The smallest doubt can stop me from acting. | 10 |  | 0.842 | 0.552 |
| When I am uncertain, I can’t function very well. | 7 |  | 0.767 | 0.661 |
| Uncertainty keeps me from living a full life. | 3 |  | 0.421 | 0.499 |
| I can’t stand being taken by surprise. | 9 |  | 0.412 | 0.465 |
| **Total variance explained** |  | 42.45% | 9.34% | 51.78% |
| **Cronbach alpha = 0.874** | | | | |
| **Kaiser-Meyer-Olkin (KMO) = 0.908** | | | | |
| **Bartlett’s test of sphericity p < 0.001** | | | | |

**Supplement Tables 7, 8 and 9** Describes the mediation analysis conducted on the impact of intolerance of uncertainty score (Table7), maladaptive coping (Table 8) and adaptive coping (Table 9) on well-being. The anxiety scale and depression scales were used separately as mediators.

| **Supplement Table 7 Mediating effect of anxiety and depression on wellbeing in terms of intolerance of uncertainty** | | | | | | |
| --- | --- | --- | --- | --- | --- | --- |
| **Model 1: Linear regression taking anxiety as dependent variable and intolerance of uncertainty as independent variable** | | | | | **Indirect effect of intolerance of uncertainty on wellbeing (anxiety as mediator)** | |
| **Factor** | **US Beta** | **SD Beta** | **95% CI)** | **P value** |  |  |
| **Intolerance of uncertainty** | 0.421 | 0.365 | 0.321; 0.522 | <0.001* | **US Beta** | **95% CI** |
| **Model 2: Linear regression taking wellbeing as dependent variable and intolerance of uncertainty and anxiety as independent variables** | | | | |  |  |
| **Factor** | **US Beta** | **SD Beta** | **95% CI)** | **P value** | -0.120 | -0.167; -0.076 |
| **Intolerance of uncertainty** | 0.106 | 0.09 | -0.006; 0.217 | 0.06 |  |  |
| **anxiety** | -0.001 | -0.327 | -0.428; -0.234 | <0.001* |  |  |
| **Model 3: Linear regression taking wellbeing as dependent variable and intolerance of uncertainty as independent variable** | | | | |  |  |
| **Factor** | **US Beta** | **SD Beta** | **95% CI)** | **P value** |  |  |
| **Intolerance of uncertainty** | -0.033 | -0.029 | -0.142; 0.075 | 0.543 |  |  |
| **Model 4: Linear regression taking depression as dependent variable and intolerance of uncertainty as independent variable** | | | | | **Indirect effect of intolerance of uncertainty on wellbeing (depression as mediator)** | |
| **Factor** | **US Beta** | **SD Beta** | **95% CI)** | **P value** |  |  |
| **Intolerance of uncertainty** | 0.188 | 0.284 | 0.129; 0.247 | <0.001* | **US Beta** | **95% CI** |
| **Model 5: Linear regression taking wellbeing as dependent variable and intolerance of uncertainty and depression as independent variables** | | | | | -0.137 | -0.190; -0.085 |
| **Factor** | **US Beta** | **SD Beta** | **95% CI)** | **P value** |  |  |
| **Intolerance of uncertainty** | 0.103 | 0.088 | 0.003; 0.204 | 0.043* |  |  |
| **Depression** | -0.849 | -0.482 | -1.001; -0.690 | <0.001* |  |  |
| **Model 6: Linear regression taking wellbeing as dependent variable and intolerance of uncertainty as independent variable** | | | | |  |  |
| **Factor** | **US Beta** | **SD Beta** | **95% CI)** | **P value** |  |  |
| **Intolerance of uncertainty** | -0.056 | -0.048 | -0.165; 0.052 | 0.306 |  |  |
| *significant values | | | | | | |

| **Supplement Table 8. Mediating effect of anxiety and depression on wellbeing in terms of maladaptive coping** | | | | | | |
| --- | --- | --- | --- | --- | --- | --- |
| **Model 7: Linear regression taking anxiety as dependent variable and maladaptive coping as independent variable** | | | | | **Indirect effect of maladaptive coping on wellbeing (anxiety as mediator)** | |
| **Factor** | **US Beta** | **SD Beta** | **95% CI)** | **P value** |  |  |
| **Maladaptive coping** | 0.801 | 0.427 | 0.646; 0.956 | <0.001* | **US Beta** | **95% CI** |
| **Model 8: Linear regression taking wellbeing as dependent variable and maladaptive coping and anxiety as independent variables** | | | | | -0.128 | -0.173; -0.079 |
| **Factor** | **US Beta** | **SD Beta** | **95% CI)** | **P value** |  |  |
| **Maladaptive coping** | 0.044 | 0.022 | -0.144; 0.233 | 0.645 |  |  |
| **anxiety** | -0.311 | -0.299 | -0.412; -0.211 | <0.001* |  |  |
| **Model 9: Linear regression taking wellbeing as dependent variable and maladaptive coping as independent variable** | | | | |  |  |
| **Factor** | **US Beta** | **SD Beta** | **95% CI)** | **P value** |  |  |
| **Maladaptive coping** | -0.205 | -0.105 | -0.382; -0.028 | 0.023* |  |  |
| **Model 10: Linear regression taking depression as dependent variable and maladaptive coping as independent variable** | | | | | **Indirect effect of maladaptive coping on wellbeing (depression as mediator)** | |
| **Factor** | **US Beta** | **SD Beta** | **95% CI)** | **P value** |  |  |
| **Maladaptive coping** | 0.487 | 0.442 | 0.397; 0.576 | <0.001* | **US Beta** | **95% CI** |
| **Model 11: Linear regression taking wellbeing as dependent variable and maladaptive coping and depression as independent variables** | | | | | -0.222 | -0.278; -0.168 |
| **Factor** | **US Beta** | **SD Beta** | **95% CI)** | **P value** |  |  |
| **Maladaptive coping** | 0.213 | 0.109 | 0.039; 0.387 | 0.02* |  |  |
| **Depression** | -0.888 | -0.503 | -1.046; -0.729 | <0.001* |  |  |
| **Model 13: Linear regression taking wellbeing as dependent variable and maladaptive coping as independent variable** | | | | |  |  |
| **Factor** | **US Beta** | **SD Beta** | **95% CI)** | **P value** |  |  |
| **Maladaptive coping** | -0.219 | -0.112 | -0.394; -0.043 | 0.014* |  |  |
| *significant values | | | | | | |

| **Supplement Table 9. Mediating effect of anxiety and depression on wellbeing in terms of adaptive coping** | | | | | | |
| --- | --- | --- | --- | --- | --- | --- |
| **Model 14: Linear regression taking anxiety as dependent variable and adaptive coping as independent variable** | | | | | **Indirect effect of adaptive coping on wellbeing (anxiety as mediator)** | |
| **Factor** | **US Beta** | **SD Beta** | **95% CI)** | **P value** |  |  |
| **Adaptive coping** | 0.105 | 0.080 | -0.016; 0.225 | 0.088 | **US Beta** | **95% CI** |
| **Model 15: Linear regression taking wellbeing as dependent variable and adaptive coping and anxiety as independent variables** | | | | | -0.026 | -0.062; 0.012 |
| **Factor** | **US Beta** | **SD Beta** | **95% CI)** | **P value** |  |  |
| **Adaptive coping** | 0.608 | 0.451 | 0.503; 0.714 | <0.001* |  |  |
| **anxiety** | -0.337 | -0.327 | -0.418; -0.256 | <0.001* |  |  |
| **Model 16: Linear regression taking wellbeing as dependent variable and adaptive coping as independent variable** | | | | |  |  |
| **Factor** | **US Beta** | **SD Beta** | **95% CI)** | **P value** |  |  |
| **Adaptive coping** | 0.573 | 0.425 | 0.460; 0.686 | <0.001* |  |  |
| **Model 17: Linear regression taking depression as dependent variable and adaptive coping as independent variable** | | | | | **Indirect effect of adaptive coping on wellbeing (depression as mediator)** | |
| **Factor** | **US Beta** | **SD Beta** | **95% CI)** | **P value** |  |  |
| **Adaptive coping** | 0.018 | 0.023 | -0.052; 0.087 | 0.631 | **US Beta** | **95% CI** |
| **Model 18: Linear regression taking wellbeing as dependent variable and adaptive coping and depression as independent variables** | | | | | -0.011 | -0.062; 0.041 |
| **Factor** | **US Beta** | **SD Beta** | **95% CI)** | **P value** |  |  |
| **Adaptive coping** | 0.578 | 0.432 | 0.482; 0.674 | <0.001* |  |  |
| **Depression** | -0.807 | -0.462 | -0.933; -0.683 | <0.001* |  |  |
| **Model 19: Linear regression taking wellbeing as dependent variable and adaptive coping as independent variable** | | | | |  |  |
| **Factor** | **US Beta** | **SD Beta** | **95% CI)** | **P value** |  |  |
| **Adaptive coping** | 0.564 | 0.421 | 0.453; 0.675 | <0.001* |  |  |
| *significant values | | | | | | |
